# Dyslexic Children Are Confronted with Unstable Binocular Fixation while Reading

**DOI:** 10.1371/journal.pone.0018694

**Published:** 2011-04-06

**Authors:** Stephanie Jainta, Zoï Kapoula

**Affiliations:** IRIS laboratory CNRS – Assistance Publique Hôpitaux de Paris, Paris, France; Smith-Kettlewell Eye Research Institute, United States of America

## Abstract

Reading requires three-dimensional motor control: saccades bring the eyes from left to right, fixating word after word; and oblique saccades bring the eyes to the next line of the text. The angle of vergence of the two optic axes should be adjusted to the depth of the book or screen and - most importantly - should be maintained in a sustained manner during saccades and fixations. Maintenance of vergence is important as it is a prerequisite for a single clear image of each word to be projected onto the fovea of the eyes. Deficits in the binocular control of saccades and of vergence in dyslexics have been reported previously but only for tasks using single targets. This study examines saccades and vergence control during real text reading. Thirteen dyslexic and seven non-dyslexic children read the French text “L'Allouette” in two viewing distances (40 cm vs. 100 cm), while binocular eye movements were measured with the Chronos Eye-tracking system. We found that the binocular yoking of reading saccades was poor in dyslexic children (relative to non-dyslexics) resulting in vergence errors; their disconjugate drift during fixations was not correlated with the disconjugacy during their saccades, causing considerable variability of vergence angle from fixation to fixation. Due to such poor oculomotor adjustments during reading, the overall fixation disparity was larger for dyslexic children, putting larger demand on their sensory fusion processes. Moreover, for dyslexics the standard deviation of fixation disparity was larger particularly when reading at near distance. We conclude that besides documented phoneme processing disorders, visual/ocular motor imperfections may exist in dyslexics that lead to fixation instability and thus, to instability of the letters or words during reading; such instability may perturb fusional processes and might – in part - complicate letter/word identification.

## Introduction

When we read a text, typically, different eye movements are required: saccades bring the eyes from left to right, fixating word after word, while oblique saccades bring the eyes to the next line of text. In parallel, the angle of vergence of the two optic axes should be adjusted to the distance of the book or screen and - most importantly - should be maintained appropriate to the distance in a sustained manner during saccades and fixations. Such maintenance of vergence is important as it is a prerequisite for the creation of a single image of each word, i.e., it establishes that the images coming from the two eyes fall on corresponding retinal areas. In other words, binocular vision of a text requires continuously monitoring the vergence angle and ensuring that it is adjusted for proper fusion of the two retinal images. Without active and fine-tuned vergence adjustments, the fusion process might fail and, since the actual fused precept is the basic ground for higher-level processing of letter and word identification [1], [2], reading might be disturbed.

Besides the most popular theories which claim that dyslexia results from impaired phonological processes troubling linguistic analysis [3], [4], currently several theories include the idea that visual/oculomotor deficits might exist in dyslexics which perturb the fusional process that in turn establishes single percepts of the letters and words [5], [6]. Deficits are even supposed to be related to a dysfunction of the magnocellular system [7].

Maintenance of the appropriate vergence angle during reading fixations depends (i) on the quality of the binocular coordination of the preceding saccade and (ii) on the disparity-driven, fine-tuning vergence movements acting during fixations to adjust the eyes properly for fusion. Although obviously dependent on each other, these two different aspects of binocular coordination give different information about vergence adjustments. Research to date has not evaluated both aspects of binocular co-ordination of the eyes by dyslexic and non-dyslexic children during naturalistic reading – neither as single entities nor in their obvious combination. Nevertheless, several studies provide basic observations about binocular coordination during saccades and fixations in non-reading tasks – and sometimes include information about dyslexic children as well. We will summarize these findings briefly in the following section.

Generally, when moving the eyes across the text, each saccade inherits a disconjugacy (a transient vergence eye movement) which is due to a difference in the sideway movements of the two eyes: typically, the abducting eye makes a larger and faster movement than the adducting eye at the beginning of the saccade [8], [9], [10], [11], [12], [13], [14]. This saccade disconjugacy was also found to be present during reading [15]. In dyslexic children, the saccade disconjugacy was found to be increased when compared with age-matched non-dyslexics in a single word reading task [6] and this observation held for free explorations of paintings [16]. Moreover, the saccade disconjugacy is typically followed by a disconjugate drift during the subsequent fixation, which passively restores the disconjugacy due to saccade, i.e., a pulse-slide-step activity recorded in abducens neurons [17]. A time analysis showed that the disconjugate drift during fixations is high at the beginning of fixations and negligible after about 50 to 100 ms [15], [18]; thus, the oculomotor system overcomes asymmetric pulse-step mismatches first during fixations, whereas after a certain time window such mismatches are resolved and the eyes reach an approximately stable vergence angle.

During reading, the eyes perform a stereotyped pattern of vergence adjustment: the saccade disconjugacy is usually divergent and followed by a convergent drift at the beginning of fixations [6], [15], [19]. However, such a pattern was not present in dyslexic children when performing a word reading task [6] or free image exploration [16]. It remains to be seen whether this holds for text reading as well. As soon as the oculomotor adjustments due to the saccade are finished, disparity-driven vergence maintenance takes place: a sensory driven, fine-tuning vergence adjustment which has to keep the images of words and letters (coming from the two eyes) stable and fused while fixating. The latter mechanism takes place within Panum's area, i.e., a small range of disparity where sensory fusion of the two retinal images is performed, thus avoiding double vision [9], [20].

There are reports that increased fixation disparities coincide with fatigue and eye strain at near vision [21], [22]. More generally, fixation disparity might be related to the resting state of the vergence system and/or the coupling of accommodation and vergence [20], [23]. There are recent reports of fixation disparities during reading, including in children (see for example, Blythe et al [19] or Kirkby et al. [24] for a review); however, regarding dyslexia, only Jaschinski et al. [25] and Cornellisen et al. [5] have reported that the amount of fixation disparity was not different between dyslexic and age-matched, non-dyslexic children when performing a simple fixation task or a single word reading task, respectively.

Generally, the variability of fixation disparity during fixations provides information on the quality of oculomotor vergence adjustments correcting the disconjugacy of the preceding saccade at the beginning of fixations, and afterwards on the quality of the sensory driven feedback loop stabilizing or improving further the vergence adjustment. The variability of the overall fixation disparity during fixations was the subject of only a few previous studies and none of them included real text reading: Eden et al. [26] reported poor vergence control and unstable fixation after saccades to single targets in dyslexic children; poor binocular control during prolonged target fixation has been also reported by Stein and Fowler [27], who used a clinical test (Dunlop test) for dyslexia assessment. The Dunlop test was invented to test eye dominance or more specifically, to test for “hemispheric dominance” within the central parts of a binocularly fused image; children with an undeveloped reference eye were supposed to be worse in reading. Some studies [5], [25], [27] suggest a correlation with fixation disparity and its stability but this remains controversial. Jaschinski et al. [25] also showed increased variability of fixation disparities during fixations to single targets, when fixation disparities were measured using psychophysical methods. More interestingly, Cornelissen et al. [5] reported no difference in the stability of the vergence error between children who passed and who failed the Dunlop test – and these children read single words at a close distance while the eye movements were recorded using an eye tracking system.

The present study aims to evaluate the maintenance of the appropriate vergence angle for dyslexic and non-dyslexic children - for the first time measured during real, text reading, i.e., when the eyes move in a sequence of fixation across a text. If defined visual/oculomotor deficits during the maintenance of fixations exist in dyslexics, these might perturb the fusional process [5], [6], [7] and it would be helpful to distinguish which aspect of binocular coordination (whether the yoking of the saccade or the sensory-driven fine tuning during fixation) is malfunctioning while reading. We included two reading distances, since fixation disparity and the binocular coordination of saccades might be different for different viewing distances for children (see for example, Jaschinski et al. [28] or [13], [14]). Including a close, typical reading distance, was especially important as it put the highest demands on vergence adjustments in terms of adjusting and keeping constant an appropriate vergence angle during successive reading saccades and fixations to ease the fusional process.

## Methods

### Ethics statement

The investigation adhered to the principles of the Declaration of Helsinki, and was approved by a local internal ethics committee for human experimentation (CPP II de France II; No 07035; Hospital Necker, Paris). Informed oral consent was obtained from each child and his or her parents after explanation of the procedure of the experiment.

### Participants

Thirteen dyslexic children officially classified as dyslexic by specialized schools, medical centres or children's hospital services were examined. The classification evaluated their dyslexia state, with an extensive examination including neurological/psychological and phonological capabilities, made in the current year of the present study. For each child, the speed of reading, the text comprehension, and the capacity for reading words/pseudowords has been evaluated by using the L2MA battery [29]. This is the standard test developed by the applied psychology centre of Paris, and commonly used in France. It includes phonological fluency tests, a visual naming task, assessing the passive lexical stock, reading irregular words and spelling tasks. Generally, the ability to use phonetic skills to decode words is assessed using the pseudoword reading test within the L2MA battery. Inclusion criteria were: (1) scores of reading abilities directly leading to a classification as dyslexic, i.e. scores in the L2MA test beyond 2 standard deviations; (2) a normal mean intelligence quotient, stated in the written report, and (3) no neurological symptoms or ophtalmological pathology. The mean age of the dyslexic children was 11.7±2 years on average for the 10 boys and 3 girls. Seven quasi aged-matched control children (4 girls, 3 boys; mean age: 12.7±1 years) were recruited mostly among children of colleagues. These control-group children had to satisfy the following criteria: (1) no history of reading difficulty, (2) no neurological symptoms or ophtalmological pathology, and (3) no visual stress or difficulties with near vision.

All children had normal or corrected-to-normal vision. Binocular vision was assessed the day of eye movement measurements as stereo-thresholds based on disparity detection via the TNO random dot test (Netherlands Organisation of Applied Scientific Research Test of stereoacuity); all individual scores were normal (60 seconds of arc or better).

### Task and stimuli

The child was seated comfortably in an adapted chair and the head stabilized with a chin rest. He/she viewed binocularly the TFT screen on which the text “L'alouette” (in french) appeared, in black letters on white background. The “L'alouette” is commonly used in France for the evaluation of reading capacity in dyslexia. It contains non-frequent words and the order of the words is unusual in French; the reader cannot use anticipation [30]. The text was written in Times New Roman (in font size 12) and each letter was about 0.3 deg of angular size. Six text panels (heights×width) of 8×10 deg were presented in sequence on the screen, covering the complete “L'alouette” text. Each panel contained 8 lines of text, double spaced. The child was asked to read the text silently but to indicate when he/she had finished reading the panel so that it could be changed into the next one. To ensure that subjects actually read the text, they were asked to briefly comment on it. In common with adults [15] the children complained about the strangeness of the text and quoted few words or parts of the context.

The children had to read the “L'alouette” at two viewing distances: close (40 cm) and far (100 cm). The text size was re-scaled according to the distance and the sequence of the text presentations was counterbalanced across children.

### Apparatus and calibration

Eye movement data for the left and right eye were measured dynamically (200 Hz) using a head-fixed (i.e., head mounted) infrared video eye tracker (Chronos Vision, Berlin); a chin rest was used to stabilise the children's head. The Chronos eye tracking system records digital image sequences and evaluates offline eye position changes with a reported resolution of less then 0.1 deg.

Before each reading block, a standard saccadic paradigm was used to elicit visually guided saccades: a target (two segments 0.9×0.7 deg, aligned vertically, with offsets of 0.1 deg vertically and 0.7 deg horizontally) jumped between five positions on the screen (at the centre and at ±8 deg horizontally and vertically). The subject was asked to follow accurately the centre of the target (at the offset space) and stable fixation periods between saccades were used to extract the calibration factors separately for each eye. Viewing during calibrations was monocular (i.e., one eye was occluded with a patch) and each saccade target was presented 4 times for each eye.

### Data analysis

Calibration and analysis methods were similar to those used in prior studies [15]. Briefly, a linear function was used to transform eye position signals into degrees. From the separate signals of the two eyes we calculated the conjugate eye movement [(left eye+right eye)/2; i.e. the version signal] and the disconjugate eye movement [left eye – right eye; i.e. the vergence signal]. We also derived the vertical conjugate signal (mean of the two vertical eye positions). The onset, or offset, of horizontal saccades were defined as the time when the eye velocity of the conjugate signal exceeded, or dropped below, respectively, 10% of the maximum velocity.

**Figure 1:**
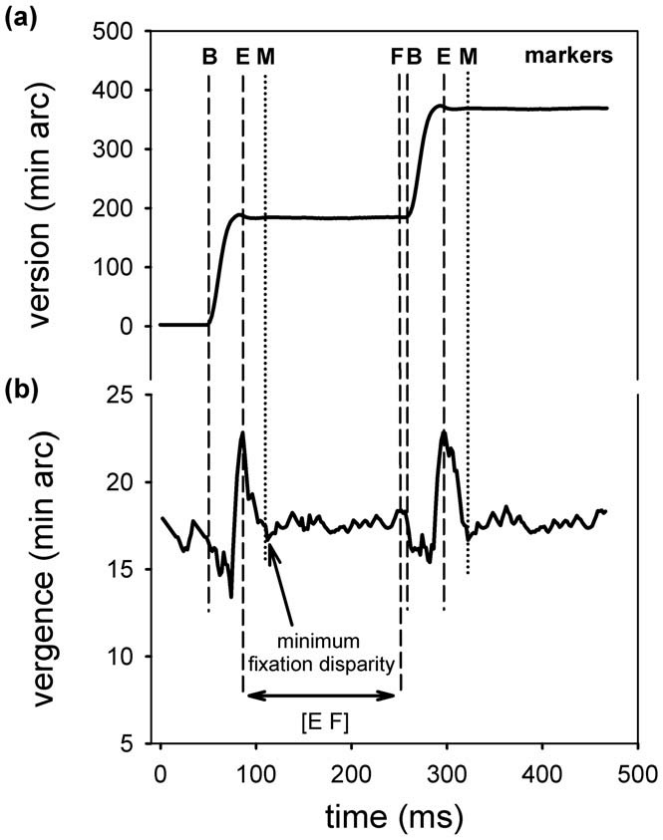
We selected a sequence of two saccades from the eye movement measurements to illustrate the placement of the markers. In (a) the version signal ((right eye+left eye)/2; min arc) is shown and two saccades can be detected easily; the saccade start was marked by an B and the end by an E. Further, the end of a fixation period was marked by an F and this end was defined as 10 ms before the next saccade started. All markers from the version signal were transferred into the vergence signal as well. In (b) the vergence signal (left eye – right eye; min arc) is shown. Additionally, for each fixation period the minimum fixation disparity was marked by an M; the interval [E F] in the vergence signal was also used to calculate the standard deviation of fixation disparity during this fixation period.

We extracted several parameters from the eye-movements signal. We will explain each of them in detail below. For each detected saccade we calculated its amplitude as difference in the version signal between the ending of the saccade (E) and its beginning (B) (markers (E-B) in Figure 1a). More importantly, we extracted the change in vergence between saccade on- and offset [6], [15], i.e., its disconjugacy as difference in the vergence signal between the markers (E-B) in Figure 1b.

Further, knowing the saccade on- and offsets, we defined fixation periods between saccades as real fixations as long as they were longer than 80 ms and shorter than 2 s. The end of a fixation period was marked by an F and this end was defined as 10 ms before the next saccade started (see Figure 1a). For each fixation period we calculated (1) the absolute minimum amount of the binocular fixation error, i.e., the minimum fixation disparity, which was marked by an M in the vergence eye movement signal [18]; in other words, we searched the fixation period, beginning at the marker E up to the end of the fixation period in order to find the moment in time at which the vergence error in respect to the actual viewing distance was smallest (see Figure 1b), thus, the vergence adjustments were most efficient for this fixation period. We knew from previous studies that this moment is mostly reached during the first 100 ms of each fixations [15], [18]: further, this measure gives an estimation of the overall shift of the fixation plane relative to the physical distance of the display on which the text was presented. Since the prevalence of more crossed (eso) or uncrossed (exo) fixations during reading is still a topic of ongoing research (see for example Liversedge et al [31], Nuthmann & Kliegl [32] or Jainta et al. [18]), a description and analysis of only the absolute amounts of fixation disparity is reported here, ignoring whether the eyes fixate in front or behind the text and being interested only on how much the eyes are crossing away for the physical screen plane where the text was displayed. (2) For the fixation period, we also calculated the disconjugate drift in vergence [6], that is, the change in vergence between the beginning of the fixation period and the minimum fixation disparity (M-E in Figure 1b). (3) last but not least, we calculated the standard deviation of the fixation disparity across the whole fixation period, i.e. across the period between the markers [E F] (as Cornelissen et al. [5] did before). This measure gave us a summed estimation of vergence adjustments and stability during fixations. Additionally, in order to check for fixation times, we calculated the fixation duration for each fixation period [E F]. We included only forward fixations into our analysis, while regressions were counted for each child. Further, for all extracted parameters we calculated the coefficient of variation (CV), which is a normalized measure of the dispersion of a probability distribution. It is defined as the ratio of the standard deviation to the mean. It is a useful measure when comparing data sets with different units or widely different means, since the standard deviation of data set is always best understood in the context of its mean.

## Results

### Reading characteristics: number of fixations, fixation durations and saccade amplitudes

Before reporting binocular control aspects we provide the results of reading parameters such as fixation durations and saccade amplitudes, which were in line with previous research [1], [24], [33]: all children made about 280 (±55) fixations while reading 6 panels of text, on average, with dyslexic children fixating slightly more often (about 15%). The number of regressions was higher for the dyslexic children (about 35% of all fixations) compared with the non-dyslexic children (about 25% of all fixations). Furthermore, average saccade amplitudes were slightly larger for dyslexic than for non-dyslexic children (2.42 deg (±0.99) and 2.09 deg (±0.52), respectively; F_1,18_ = 3.22, p = 0.08) but distance had no significant effect on saccade amplitudes (F value <1). The same was true for fixation durations, i.e. dyslexic children fixated slightly longer than non dyslexic children (351.0 ms (±118.4) vs. 280.1 ms (±85.1), respectively; F_1,18_ = 3.25, p = 0.09), but viewing distance did not modulate this difference (F value <1).

### Saccade disconjugacy and the disconjugacy drift during fixation

Our study replicated the previously described effect [6], that dyslexic children show larger saccade disconjugacies than typically reading children (F_1,18_ = 15.66, p<0.01); but, saccade disconjugacy did not change with reading distance for both groups of children (F_1,18_ = 1.69) – neither was the saccade disconjugacy for only one group affected by viewing distance (F-value <1). Table 1 summarizes the means, standard deviations and ranges for all 4 parameters of binocular coordination during reading.

**Table 1:** Average values, standard deviations, ranges and variability coefficients (CV) for (a) saccade disconjugacy, (b) disconjugate drift during fixations, (c) fixation disparity and (d) its standard deviation within fixations.

(a)	saccade disconjugacy (deg)							
	close (40 cm)				far (100 cm)			
group	mean	(SD)	range	CV	mean	(SD)	range	CV
non-dyslexics(n = 7)	**0.12**	(0.05)	0.10–0.23	**0.42**	**0.14**	(0.06)	0.07–0.27	**0.43**
dyslexics(n = 13)	**0.23**	(0.10)	0.12–0.36	**0.44**	**0.20**	(0.08)	0.10–0.33	**0.40**

(b)	disconjugate drift during fixation (deg)							
	close (40 cm)				far (100 cm)			
group	mean	(SD)	range	CV	mean	(SD)	range	CV
non-dyslexics(n = 7)	**0.11**	(0.02)	0.07–0.13	**0.18**	**0.11**	(0.05)	0.07–0.17	**0.45**
dyslexics(n = 13)	**0.13**	(0.10)	0.03–0.20	**0.76**	**0.14**	(0.08)	0.04–0.18	**0.57**

(c)	fixation disparity (deg)							
	close (40 cm)				far (100 cm)			
group	mean	(SD)	range	CV	mean	(SD)	range	CV
non-dyslexics(n = 7)	**0.38**	(0.17)	0.16–0.56	**0.45**	**0.29**	(0.08)	0.06–0.51	**0.28**
dyslexics(n = 13)	**0.43**	(0.28)	0.18–0.62	**0.65**	**0.38**	(0.14)	0.18–0.67	**0.37**

(d)	standard deviation of fixation disparity (deg)							
	close (40 cm)				far (100 cm)			
group	mean	(SD)	range		mean	(SD)	range	
non-dyslexics(n = 7)	**0.16**	(0.05)	0.11–0.22		**0.10**	(0.01)	0.07–0.11	
dyslexics(n = 13)	**0.41**	(0.20)	0.12–0.77		**0.26**	(0.14)	0.12–0.48	

**Figure 2:**
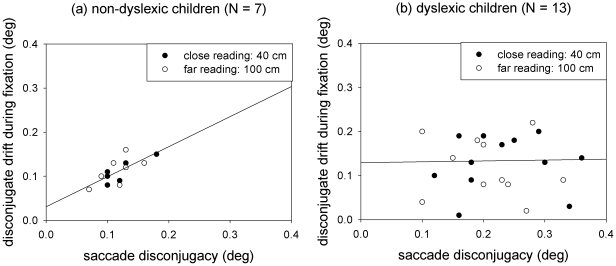
The average conjugate drift during fixations (deg) as a function of the saccade disconjugacy (deg) for non-dyslexic (a) and dyslexic (b) children.

Further, the disconjugate drift during fixations, that is mainly used to compensate for the remaining disconjugacy which occurred during saccades, was not different for non-dyslexic and dyslexic children (F value <1). Moreover, it also did not change with viewing distance (F value <1). When comparing the saccade disconjugacy and the disconjugate drift during fixations across children within each group, non-dyslexics showed a clear correlation of both measures (r = 0.71; p<0.01), while non-dyslexics did not (r = 0.10) – as expected from previous reports. Figure 2 shows the correlations of the saccade disconjugacy and the disconjugate drift during fixations for the two groups.

### Fixation disparity and the standard deviation of fixation disparity

Interestingly, analyzing the horizontal fixation disparity showed a tendency for an increased fixation disparity for close reading (F_1,18_ = 3.43, p = 0.08) and a clear tendency for an overall larger fixation disparity for dyslexic children (F_1,18_ = 3.68, p = 0.07), while these effects did not interact (F-value <1).

**Figure 3:**
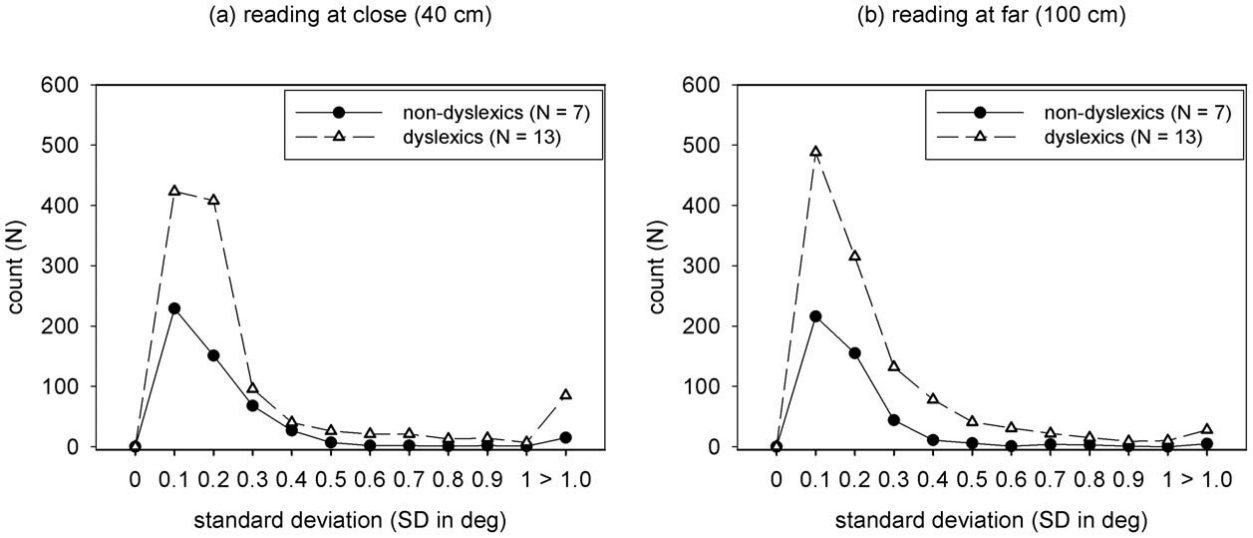
Histogram of the standard deviation (SD in deg) of fixation disparity measured while the children read the text at (a) close reading distance (40 cm) and at (b) far reading distance (100 cm); the plots show the data for non-dyslexic (dots) and dyslexic children (triangles), respectively.

In addition, we found a significant difference between non-dyslexic and dyslexic children in the stability of the fixation disparity during reading fixations (F_1,18_ = 11.60, p<0.01). Furthermore, for both groups of children, the shortening of the reading distance increased the variability of fixation disparity (F_1,18_ = 4.27, p = 0.05) and this increase was more pronounced in dyslexic children (F_1,18_ = 6.73, p = 0.02). Figure 3 shows the probability distributions for the standard deviation of fixation disparity, while the means and ranges can be found in Table 1.

We wondered if the standard deviation of fixation disparity, i.e., the variability in fixation disparity during fixations, was dependent on the position within the text. For that reason we plotted the standard deviations as function of horizontal and vertical fixation position – reflecting where on the screen the child was looking at.

**Figure 4:**
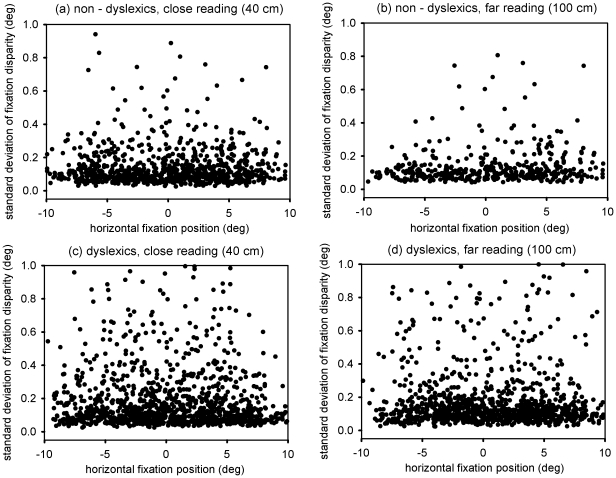
The different plots show the standard deviation of fixation disparity as a function of horizontal fixation position (deg), i.e., as a function of the horizontal position within the text at which the children looked at. The two lefthand plots show data for the close reading while the two righthand plots show data for the far reading. Upper plots (a & b) are those for non-dyslexic children (N = 7) while the lower plots (c & d) show data of the dyslexic children (N = 13).

**Figure 5:**
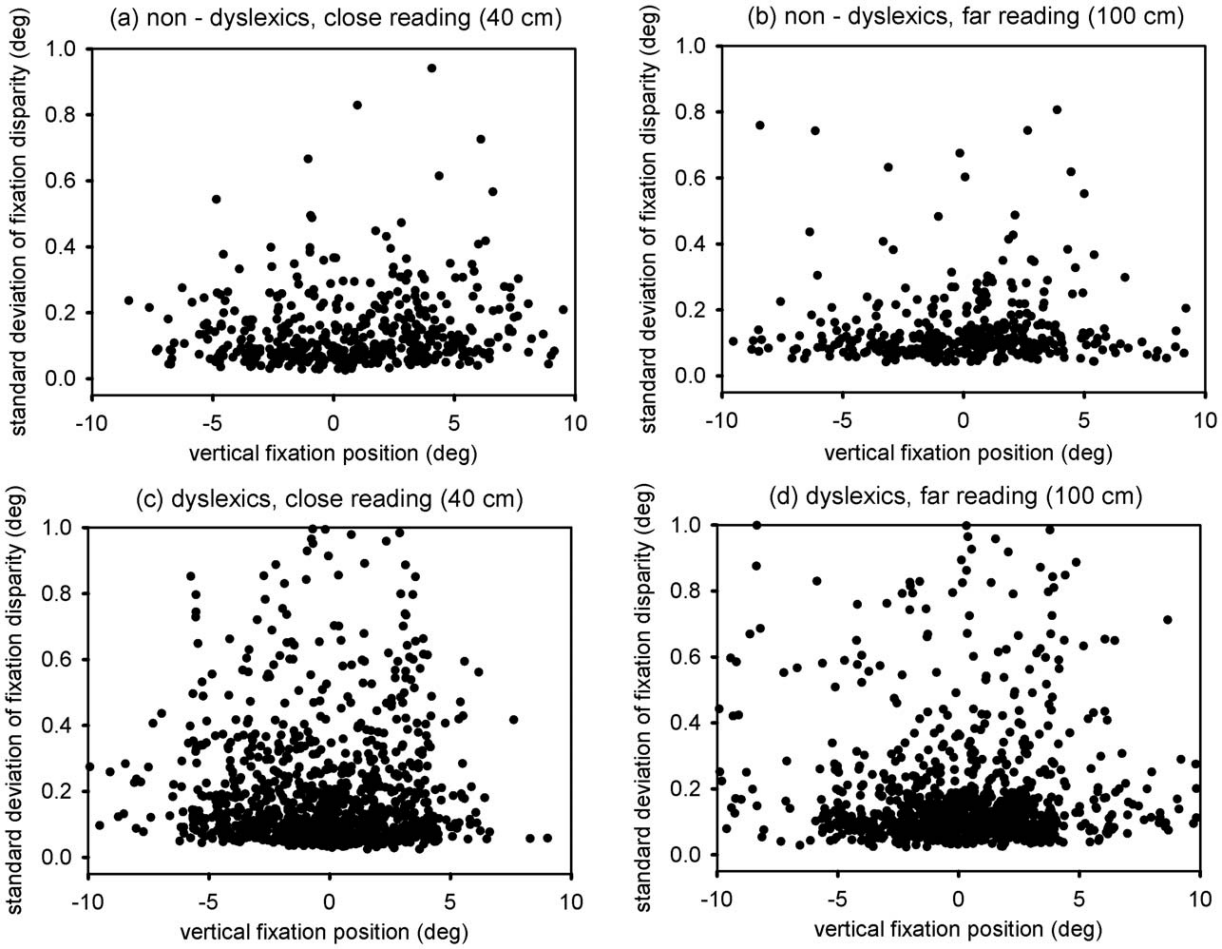
The different plots show the standard deviation of fixation disparity as a function of vertical fixation position (deg), i.e., as a function of the vertical position within the text at which the children looked at. The two lefthand plots show data for the close reading while the two righthand plots show data for the far reading. Upper plots (a & b) are those for non-dyslexic children (N = 7) while the lower plots (c & d) show data of the dyslexic children (N = 13).

As can be seen in Figure 4 and Figure 5, there was no obvious dependency of the standard variation of fixation disparity on the fixation position – neither for non-dyslexic nor for dyslexic children. We checked the data statistically by running separate linear regression analysis for the two groups (statistical package R [34]), including the effects of the fixation position (horizontal and vertical) and the viewing distance. Besides the already described effect of the viewing distance (t = 4.01 for non-dyslexics and t = 4.46 for dyslexics, respectively) none of the fixation positions had a significant influence on the standard deviation of fixation disparity (all t-values for the beta-weights ≤1).

### The variability coefficient (CV)

As can be seen in Table 1, the coefficients of variability range between 0.12 and 0.74 for the different extracted oculomotor parameters during reading. In most cases these coefficients were larger for dyslexic children when compared with the non-dyslexic children. Especially for the description of fixation disparity, the CVs reflected the effects of increased variability for dyslexics and this was most prominent for the close reading distance (40 cm). It is interesting to note that the CVs for the saccade disconjugacy were about the same for both distances and for dyslexic and non-dyslexic children. By contrast, the CVs changed dramatically for the disconjugacy drift during fixations. In other words, the difference in the saccade disconjugacy between both groups reflected only that the saccadic disconjugacy was simply larger in absolute values for dyslexic children, while for the disconjugacy drift during fixations a larger variability could only be observed for dyslexic children.

## Discussion

### Summary of the results

The data showed that there were more fixations and more regressions, as well as a tendency for larger saccade amplitudes for dyslexic children compared with non-dyslexic children. Together with the observation of slightly longer fixation durations in dyslexics, all these results are in line with previous reports (see, for example Pavlidis [33], and for reviews Kirkby et al. [24] or Rayner [1]). The novel results are an increased saccade disconjugacy in dyslexics, increased disconjugate post-saccadic drift, and the uncorrelated saccade and post-saccadic drift disconjugacy occurring also during text reading; these observations extend prior studies using simple target tasks or free exploration of images [6]. In absolute values, the passive disconjugate drift at the beginning of fixations after the saccade was not big enough to counterbalance the saccade disconjugacy in dyslexic children. Regarding the sensory driven, fine tuning of vergence adjustment during fixations, the overall fixation disparity showed a tendency for slightly larger fixation disparities for dyslexic children and a general tendency for larger fixation disparities for reading at near distance for all children. However, the most important result is that dyslexic children showed larger standard deviation of their fixation disparity during fixations than non-dyslexic children, and that this effect was more pronounced for the close reading distance, reflecting a remarkable demand on fusional processes to obtain single clear vision of the words. It should be noted that the close reading distance increased the standard deviations of fixation disparity even for non-dyslexic children, but not by as much as for dyslexic children. We will discuss all these different aspects in detail below.

### Saccade disconjugacy and the disconjugate drift during reading fixations

Regarding the oculomotor adjustments of vergence during reading, we showed in concordance with Bucci et al. [6] and Kapoula et al. [16] – but this time for a typical reading task, in which the children made a sequence of saccades and fixations - that the binocular coordination during and after saccades is poor in dyslexic children compared to non-dyslexics of quasi-matched age. Saccade disconjugacy is larger in dyslexics and this was found to be the case regardless of the reading distance. Recall that previous reports (using non-reading tasks) showed that the disconjugacy of saccades (and the related disconjugate drift during fixations) drops to the small values seen in adults around the age of 11 to 12 years and no viewing distance effect could be observed anymore [13], [14]. The present study indicates that in dyslexics of that age the disconjugacy deficit is still present and for both distances; this observation is new and opposite to our initial expectation that conjugacy behaviour might had been normal at that age for the far distance. Presumably there is resistant saccade and fixation disconjugacy during text reading regardless of the viewing distance.

Typically the saccade disconjugacy is followed by a disconjugate drift during the subsequent fixation, which passively restores the disconjugacy due to saccade, i.e. a pulse-slide-step activity recorded in abducens neurons [17]. We also found that the stereotyped pattern of the vergence during and after the saccade is missing for dyslexics during a text reading task: the disconjugacy occurring during the saccade is not corrected by the subsequent disconjugate drift during fixation. This result extends prior reports [6]. The origin and the importance of such a stereotyped pattern are still discussed [11], [16], [35], [36], but a reduced saccade–vergence adaptive mechanism could be responsible for the poor yoking of saccades in dyslexics, given that their divergence movements are significantly reduced relative to non-dyslexics when clinically tested [6], [16], [37]. It is important to note that a missing correlation between the disconjugacy of their saccades and the disconjugate drift during fixations reflects that the vergence adjustments did not work in a systematic manner, thus, causing a substantial variability of the vergence angle during reading fixations.

### Fixation disparity and the standard deviation of fixation disparityin reading

The sensory driven, fine-tuning vergence adjustment while fixating should provide and maintain basic grounds for the fusional process to establish a single percept. The better the vergence adjustments the less the sensory, fusional processes have to cope with a residual fixation disparity, which as mentioned can be associated with vision fatigue and eye strain [21], [22]. We found a slight tendency for larger fixation disparities in dyslexics while reading a real text – in contrast to Jaschinski et al. [25] or Cornelissen et al. [5]. In other words, dyslexic children have to handle - by means of sensory compensation - slightly larger residual disparities when actually fusing the images of the text coming from both eyes. This might cause some fatigue or just put stress on fusional capacities while reading. Additionally, the fixation disparity was slightly increased when reading was done at a close viewing distance but this time for all children; this is in line with previous research showing some dependency of fixation disparities on viewing distances for non-reading conditions [38], [39].

In addition to larger mean fixation disparities in dyslexics, our study clearly showed, for the first time, that the standard deviation of fixation disparity during reading fixations were increased in dyslexic children. This variability puts an additional demand on the fusional processes since the fusional system has to compensate for changing disparities for the same letter or word. Such variability might complicate letter or word identification processes [5], [6], [7] and supports the suggestion that - besides impaired phonological processes – a visual/ocular motor deficits exist in dyslexics which might perturb the fusional process [5], [6], [7], [27]. Furthermore, in our study the increased variability of fixation disparity in dyslexics was pronounced even for close reading conditions, which might have practical implications when designing the best reading conditions for dyslexic children: close distances increase the demand on fusional processes so that letter or word identification processes might be even more difficult to accomplish [5], [6], [7].

### Fixation disparity and its standard deviation during free exploration of a painting

A question arises concerning the general nature of the increased standard deviation of fixation disparity: is it found only in reading tasks only or also in non-reading tasks? To address this question, we ran a short follow up study with children, who had already participated in the reading study. For 2 non-dyslexics (aged 13 and 14 years) and 3 dyslexic (aged 13, 16 and 12 years) children we added an additional task to the experimental design: after having read the “L'alouette” text at two distances, these children freely explored the unrealistic cubist painting “The Alarm Clock” by Fernand Leger for 30 sec [16]. The eye movement signal of the Chronos Eye Tracking system was newly calibrated (monocularly) before each of these two short presentations and we extracted saccade amplitudes, fixation durations, saccade disconjugacies, the discongugate drift in veregnce during fixations as well as fixation disparities and their standard deviations for these presentations as described above (see Methods section). The pictures were 10×7 deg large (heights×width) and re-scaled according to the presentation distance (40 cm vs. 100 cm).

**Figure 6:**
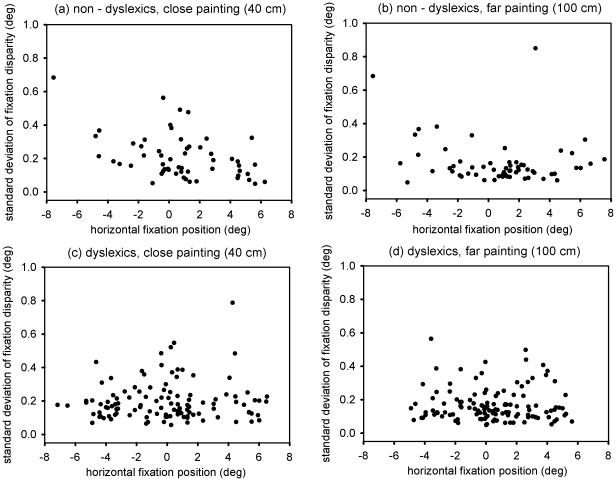
The different plots show the standard deviation of fixation disparity as a function of horizontal fixation position (deg), i.e., as a function of the horizontal position within the painting which the children looked at. The two lefthand plots show data for a close viewing distance while the two righthand plots show data for the far viewing distance, respectively. Upper plots (a & b) are those for non-dyslexic children (N = 2) while the lower plots (c & d) show data of the dyslexic children (N = 3).

Table 2 shows the means and standard deviations for all 5 children; the children made between about 70 and 150 fixations within the 30 sec and this time all fixations where analysed. Further, we plotted the standard deviation of fixation disparity as function of horizontal fixation position (see Figure 6). As can be seen in Table 2 and Figure 6, there is a slight tendency that the dyslexic children showed larger standard deviations for fixation disparity - even while freely exploring a painting. Future research will show if this increased standard deviation for dyslexic children in close viewing distances is a general aspect of their binocular coordination. As can be seen in this follow up study, the data suggest that the standard deviations of fixation disparity are further increased during free exploration of the painting – as well as the disconjugacy of the saccades; the latter confirms prior observations from [16].

**Table 2:** Means and standard deviations (SD) for 2 non-dyslexic and 3 dyslexic children while freely exploring a painting for 30 sec.

	distance to painting (40 cm)					distance to painting (100 cm)				
	non-dyslexic		dyslexic			non-dyslexic		dyslexic		
variable/child	C1	C2	D1	D2	D3	C1	C2	D1	D2	D3
saccade amplitude (deg)	1.48 (1.12)	1.33 (1.19)	0.90 (0.83)	1.41 (1.07)	1.35 (0.75)	1.36 (0.76)	1.86 (0.81)	1.77 (0.61)	0.87 (0.81)	1.62 (0.77)
saccade disconj. (deg)	0.18 (0.15)	0.19 (0.13)	0.24 (0.10)	0.23 (0.16)	0.27 (0.17)	0.21 (0.16)	0.10 (0.04)	0.33 (0.20)	0.25 (0.17)	0.37 (0.25)
disconjugate drift (deg)	0.18 (0.15)	0.19 (0.13)	0.24 (0.10)	0.23 (0.16)	0.27 (0.17)	0.21 (0.16)	0.10 (0.04)	0.33 (0.20)	0.25 (0.17)	0.37 (0.25)
fixation duration (ms)	284.1 (160.4)	384.6 (192.3)	183.7 (99.8)	279.6 (99.3)	379.0 (193.2)	272.4 (134.9)	365.0 (160.2)	268.7 (179.3)	369.4 (163.7)	231.1 (168.7)
fixation disparity (deg)	0.26 (0.53)	0.30 (0.32)	0.49 (0.46)	0.27 (0.53)	0.50 (0.53)	0.20 (0.48)	0.18 (0.32)	0.37 (0.31)	0.27 (0.21)	0.46 (0.58)
SD of fixation disparity (deg)	0.22 (0.12)	0.17 (0.13)	0.61 (0.20)	0.18 (0.16)	0.32 (0.76)	0.23 (0.13)	0.16 (0.13)	0.38 (0.24)	0.24 (0.23)	0.22 (0.21)

Further research is needed to understand the failure component but also the eventual functional aspects of such fixation instability in dyslexics that is generalized in image exploration. Fixation instability might be harmful for perception of images and could even contribute to perception of virtual, pictorial depth or pictorial movement but this needs further investigation. We argue that increased fixation instability during reading might interfere with fusional processes, but that the consequences of such a binocular instability for reading speed and reading comfort still have to be quantified.
